# IL-1RN VNTR Polymorphism in Adult Dermatomyositis and Systemic Lupus Erythematosus

**DOI:** 10.1155/2014/953597

**Published:** 2014-08-19

**Authors:** Zornitsa Kamenarska, Gyulnas Dzhebir, Maria Hristova, Alexey Savov, Anton Vinkov, Radka Kaneva, Vanio Mitev, Lyubomir Dourmishev

**Affiliations:** ^1^Molecular Medicine Center and Department of Medical Chemistry and Biochemistry, Medical University-Sofia, 2 Zdrave Street, 1431 Sofia, Bulgaria; ^2^Department of Clinical Laboratory and Clinical Immunology and Department of Nephrology, Medical University-Sofia, 1 Georgi Sofijski Street, 1431 Sofia, Bulgaria; ^3^National Genetic Laboratory, Maichin Dom Hospital, 2 Zdrave Street, 1431 Sofia, Bulgaria; ^4^28 Diagnostic and Consultative Center-Sofia, 1 Iliya Beshkov Street, 1592 Sofia, Bulgaria; ^5^Department of Dermatology and Venereology, Medical University-Sofia, 1 Georgi Sofijski Street, 1431 Sofia, Bulgaria

## Abstract

Polymorphisms in the cytokine genes and their natural antagonists are thought to influence the predisposition to dermatomyositis (DM) and systemic lupus erythematosus (SLE). A variable number tandem repeat (VNTR) polymorphism of 86 bp in intron 2 of the interleukin-1 receptor antagonist (IL-1RN) gene leads to the existence of five different alleles which cause differences in the production of both IL-1RA (interleukin-1 receptor antagonist) and IL-1*β*. The aim of this case-control study was to investigate the association between the IL-1RN VNTR polymorphism and the susceptibility to DM and SLE in Bulgarian patients. Altogether 91 patients, 55 with SLE and 36 with DM, as well as 112 unrelated healthy controls, were included in this study. Only three alleles were identified in both patients and controls ((1) four repeats, (2) two repeats, and (3) five repeats). The IL-1RN*2 allele (*P* = 0.02, OR 2.5, and 95% CI 1.2–5.4) and the 1/2+2/2 genotypes were found prevalent among the SLE patients (*P* = 0.05, OR 2.6, and 95% CI 1–6.3). No association was found between this polymorphism and the ACR criteria for SLE as well as with the susceptibility to DM. Our results indicate that the IL-1RN VNTR polymorphism might play a role in the susceptibility of SLE but not DM.

## 1. Introduction

Dermatomyositis (DM) and systemic lupus erythematosus (SLE) are diseases of unknown etiology. However, the dysregulation of cytokine production or action is thought to have an important role in their development [1].

Interleukin-1*α* (IL-1*α*) and interleukin-1*β* (IL-1*β*) are proinflammatory cytokines which belong to the IL-1 family. The interleukin-1 receptor antagonist (IL-1RA) is a naturally occurring competitive inhibitor of IL-1. The dysregulation of IL-1 production caused by IL-1RA leads to abnormal inflammatory activity which results in subsequent tissue damage commonly observed in the pathogenesis of SLE and DM. The interleukin-1 receptor antagonist (IL-1RN) gene is polymorphic, resulting in quantitative differences in both IL-1RA and IL-1*β* production. A tandem repeat sequence of 86 base pairs in length was described in intron 2 of the IL-1RN gene [2]. The number of times this sequence is repeated varies from 2 to 6. The most common is allele 1 (four repeats) followed by allele 2 (2 repeats). The other three alleles are rare, found in less than 1% in most populations. IL-1RN*2 allele was found associated with increased IL-1RA production in vitro [3]. The serum levels of IL-1RA were found significantly higher in lupus [4] and DM [5, 6] patients than in controls. The higher IL-1RA levels could serve as predictive biomarker for renal involvement in SLE [7] and positively correlated with PM/DM disease activity [8, 9].

The objective of our study was to determine whether the IL-1RN VNTR polymorphism is a risk factor for the development of adult DM and SLE in Bulgarian patients and to define its contribution to the increased risk.

## 2. Materials and Methods

### 2.1. Patient Population

Thirty-six patients with dermatomyositis who met the criteria of Bohan and Peter [10, 11] and Targoff et al. [12] and fifty-five with systemic lupus erythematosus who met the American College of Rheumatology (ACR) criteria were included in this study. Only patients with definite or probable disease were included. The clinical and demographic data are presented in Table 1. In the DM group, 23 patients were female and 13 male. The mean age was 52 with a range of 18–82 years. In the SLE group, 46 were female and 9 male. The mean age was 40 with a range of 15–78 years. The patients have been followed for a mean of 10 years at the Department of Dermatology and Venereology, Medical University-Sofia, at the Department of Nephrology, Medical University-Sofia, and at the Department of Nephrology, Ministry of Interior Hospital-Sofia.

The control group consisted of 112 anonymous healthy volunteers who did not show any clinical or laboratory signs of autoimmune skin diseases, as well as kinship with patients suffering from autoimmune skin diseases. They were randomly selected from the Biobank of the Molecular Medicine Center and the National Genetic Laboratory as to match the patients in age, gender, and ethnicity.

### 2.2. Genetic Analysis

The scientific investigation presented in this paper has been carried out in accordance with The Code of Ethics of the World Medical Association (Declaration of Helsinki) for experiments involving humans. The study was approved by the local ethics committee at the Medical University-Sofia. All participants signed an informed consent and venous blood was drawn for DNA isolation. Genomic DNA was extracted from the peripheral blood with the Chemagen DNA purification kit, using Chemagic Magnetic Separation Module I (Chemagen AG).

The analysis of IL-1RN 86 bp repeat polymorphism was performed as previously described by Tarlow et al. [2]. Standard primer pairs were used (5′-CTCAGCAACACTCCTAT; 3′-TCCTGGTCTGCAGGTAA). Amplification was performed under the following conditions: denaturing step at 95°C for 10 minutes, 35 cycles of 95°C for 60 seconds, 59°C for 60 seconds, 72°C for 60 seconds, and 1 cycle of extension at 72°C for 10 minutes. The products were visualized on a 3% agarose gel stained with ethidium bromide.

### 2.3. Statistical Analysis

Allele and genotype frequencies were compared between DM and SLE cases and controls, using Fisher's exact test to calculate *P* values for 2 × 2 tables. Where significant, data were expressed as *P* value, odds ratios (OR) with exact 95% confidence intervals (CI).

## 3. Results

The observed allele and genotype frequencies of the IL-1RN VNTR polymorphism among the patients with DM, SLE, and the healthy controls are summarized in Table 2.

Only three alleles were detected among the Bulgarian population: 1 (four repeats), 2 (two repeats), and 3 (five repeats). The IL-1RN*2 allele (*P* = 0.02, OR 2.5, and 95% CI 1.2–5.4) and the 12 + 22 genotypes (*P* = 0.05, OR 2.6, and 95% CI 1–6.3) were found associated with SLE (Table 2).

No association was found between that polymorphism and DM as well as with the clinical manifestations of the two diseases (Table 3).

## 4. Discussion

The majority of the studies relating IL-1RN gene polymorphisms to disease susceptibility have dealt with patients with autoimmune diseases or disorders associated with chronic inflammation [13].

The first study to correlate this polymorphism with SLE susceptibility was done on Caucasians and the carriage of IL-1RN*2 allele was reported to be associated with severity rather than susceptibility to SLE [14]. The association strengthened with extensive disease and particularly with the presence of photosensitivity and discoid skin lesions. The association of the IL1-RN*2 allele with SLE was confirmed for Japanese patients and it was again increased with photosensitivity [15]. Similarly, increased frequencies of malar rash and photosensitivity were observed among patients with IL1-RN*2 compared to patients without the allele [16]. In our study we have not observed any association between that polymorphism and ACR criteria. However, we have observed a higher frequency of the IL-1RN*2 allele and 1/2 + 2/2 genotype in patients with SLE which is in line with the results of most of the previous studies [14–18]. Our results correlate well with the results of a recent meta-analysis [19]. In Malaysia, however, the risk allele associated with SLE susceptibility was IL-1RN*1 [20] while other authors could not find any association between that polymorphism and SLE [21–23]. Quite surprisingly Mohammadoo-Khorasani et al. [24] found an association between the IL-1RN*4 allele and the 1RN* 1/4 genotype and the development of SLE in Iranian cohort. Such discrepancies could be attributed to interethnic variations [25], low frequency of the IL-1RN*2 allele, small sample size which lacks statistical power, or influence of other polymorphisms. Furthermore, it was shown that IL-1RA 2/2 was not individually associated with SLE but the combination of the Fc*γ*RIIa R/R and IL-1RN 2/2 genotypes is associated with SLE in Caucasian patients [26]. The studies concerning the polymorphisms in the IL-1RN gene could have practical aspects since they might affect the response towards Anakinra, which is a recombinant form of human IL-1RA [27].

The frequency of the IL-1RN*2 allele was low among the DM patients and no statistically significant associations were found in allele and genotype distribution. Our results correlate with the results of other authors [8]. Interestingly Rider et al. [28] have found the IL-1RN*1 allele being associated with juvenile idiopathic inflammatory myopathies (JIIM) in Caucasians, while the IL-1RN*3 allele was associated with JIIM in African-Americans.

In summary, our results indicate that IL-1RN VNTR polymorphism might play a role in the susceptibility of SLE but not DM in Bulgarian patients.

## Figures and Tables

**Table 1:** Demographic and clinical data.

Disease	DM		SLE	
Demographic parameters	Female/male	23/13	Female/male	46/9
	Age, mean ± SD years	52 ± 14.7	Age, mean ± SD years	40 ± 12.4

Clinical parameters	Cutaneous disease Muscle weakness Elevated muscle enzymes EMG findings Photosensitivity Autoantibodies	27 (78.8%) 28 (78.8%) 19 (54.6%) 20 (54.6%) 21 (60.6%) 9 (18.2%)	Malar rash Discoid rash Arthritis Oral ulcer Photosensitivity Serositis Renal disease Neurological disease Haematological disease Immunological disease ANA	34 (61.8%) 11 (20.0%) 37 (67.3%) 4 (7.3%) 31 (56.4%) 11 (20.0%) 55 (100%) 12 (22.2%) 20 (36.4%) 34 (61.8%) 39 (70.9%)

SD: standard deviation, EMG: electromyography, and ANA: antinuclear antibodies.

**Table 2:** Genotype and allele frequencies of the IL-1RN VNTR polymorphism among patients with DM, SLE, and controls.

Genotype	DM	SLE	Controls
*N*: number of patients	*N* = 36	*N* = 55	*N* = 112
Genotypes			
1/1	33 (91.7%)	41 (74.6%)	96 (85.7%)
1/2	0 (0.0%)	8 (14.5%)	9 (8.0%)
2/2	1 (2.8%)	4 (7.3%)	2 (1.8%)
1/3	2 (5.6)	1 (1.8%)	4 (3.6%)
2/3	0 (0.0%)	0 (0.0%)	1 (0.9%)
3/3	0 (0.0%)	1 (1.8%)	0 (0.0%)
*P* value	NS∗	**12** + **22**, **P** =** 0.05**	
Alleles			
1	68 (94.4%)	91 (82.7%)	205 (91.5%)
2	2 (2.8%)	16 (14.6%)	14 (6.3%)
3	2 (2.8%)	3 (2.7%)	5 (2.2%)
*P* value	NS	**2, ** **P** = **0.02**	

∗NS: not significant.

**Table 3:** Comparison between the genotypes and the ACR criteria for SLE.

Genotype	1/1 (*n* = 41)	1/2 (*n* = 8)	2/2 (*n* = 4)	1/3 (*n* = 1)	3/3 (*n* = 1)	*P* value
Malar rash	28 (68.3%)	4 (50.0%)	2 (50.0%)	0 (0.0%)	0 (0.0%)	NS∗
Discoid rash	7 (17.1%)	3 (37.5%)	1 (25.0%)	0 (0.0%)	0 (0.0%)	NS
Photosensitivity	23 (57.1%)	5 (61.5%)	2 (50.0%)	1 (100.0%)	0 (0.0%)	NS
Oral ulcer	3 (7.3%)	1 (12.5%)	0 (0.0%)	0 (0.0%)	0 (0.0%)	NS
Arthritis	29 (70.7%)	5 (62.5%)	2 (50.0%)	0 (0.0%)	1 (100.0%)	NS
Serositis	10 (24.4%)	1 (12.5%)	0 (0.0%)	0 (0.0%)	0 (0.0%)	NS
Renal disease	41 (100.0%)	8 (100.0%)	4 (100.0%)	1 (100.0%)	1 (100.0%)	NS
Neurological disease	10 (22.9%)	1 (12.5%)	0 (0.0%)	0 (0.0%)	1 (100.0%)	NS
Haematological disease	15 (36.6%)	4 (50.0%)	1 (25.0%)	0 (0.0%)	0 (0.0%)	NS
Immunological disease	24 (58.3%)	5 (62.5%)	3 (75.0%)	1 (100.0%)	1 (100.0%)	NS
(Anti-dsDNA, anti-Sm, anti-phospholipid Ab)						
ANA	29 (70.7%)	7 (87.5%)	2 (50.0%)	0 (0.0%)	1 (100.0%)	NS

∗Not significant, anti-dsDNA: antibodies to the double stranded DNA, anti-SM: anti-Smith antibodies (specific markers for SLE), and ANA: antinuclear antibodies.
